# Metastatic breast cancer cells overexpress and secrete miR-218 to regulate type I collagen deposition by osteoblasts

**DOI:** 10.1186/s13058-018-1059-y

**Published:** 2018-10-22

**Authors:** Xuxiang Liu, Minghui Cao, Melanie Palomares, Xiwei Wu, Arthur Li, Wei Yan, Miranda Y. Fong, Wing-Chung Chan, Shizhen Emily Wang

**Affiliations:** 1 Department of Pathology, University of California San Diego, 9500 Gilman Drive, La Jolla, CA 92093-0612 USA; 2 City of Hope Irell & Manella Graduate School of Biological Sciences, Duarte, CA 91010 USA; 3 Cancer Prevention Movement, Arcadia, CA 91006 USA; 4 Department of Molecular and Cellular Biology, Beckman Research Institute of the City of Hope, Duarte, CA 91010 USA; 5 Division of Biostatistics, Beckman Research Institute of the City of Hope, Duarte, CA 91010 USA; 6 Department of Cancer Biology, Beckman Research Institute of the City of Hope, Duarte, CA 91010 USA; 7 Department of Pathology, City of Hope National Medical Center, Duarte, CA 91010 USA

**Keywords:** Breast cancer, Bone metastasis, miRNA, Cytokines, Osteoblasts, Type I collagen

## Abstract

**Background:**

Bone is one of the most frequent metastatic sites of advanced breast cancer. Current therapeutic agents aim to inhibit osteoclast-mediated bone resorption but only have palliative effects. During normal bone remodeling, the balance between bone resorption and osteoblast-mediated bone formation is essential for bone homeostasis. One major function of osteoblast during bone formation is to secrete type I procollagen, which will then be processed before being crosslinked and deposited into the bone matrix.

**Methods:**

Small RNA sequencing and quantitative real-time PCR were used to detect miRNA levels in patient blood samples and in the cell lysates as well as extracellular vesicles of parental and bone-tropic MDA-MB-231 breast cancer cells. The effects of cancer cell-derived extracellular vesicles isolated by ultracentrifugation and carrying varying levels of miR-218 were examined in osteoblasts by quantitative real-time PCR, Western blot analysis, and P1NP bone formation marker analysis. Cancer cells overexpressing miR-218 were examined by transcriptome profiling through RNA sequencing to identify intrinsic genes and pathways influenced by miR-218.

**Results:**

We show that circulating miR-218 is associated with breast cancer bone metastasis. Cancer-secreted miR-218 directly downregulates type I collagen in osteoblasts, whereas intracellular miR-218 in breast cancer cells regulates the expression of inhibin β subunits. Increased cancer secretion of inhibin βA results in elevated Timp3 expression in osteoblasts and the subsequent repression of procollagen processing during osteoblast differentiation.

**Conclusions:**

Here we identify a twofold function of cancer-derived miR-218, whose levels in the blood are associated with breast cancer metastasis to the bone, in the regulation of type I collagen deposition by osteoblasts. The adaptation of the bone niche mediated by miR-218 might further tilt the balance towards osteolysis, thereby facilitating other mechanisms to promote bone metastasis.

**Electronic supplementary material:**

The online version of this article (10.1186/s13058-018-1059-y) contains supplementary material, which is available to authorized users.

## Background

The “Seed and Soil” hypothesis first brought up by Stephen Paget highlights the importance of matching between cancer and its metastatic niche, and implies the requirement of niche adaptation during cancer metastasis [1]. The bone tropism of breast cancer cells is in part mediated by chemokines and their receptors, exemplified by C-X-C chemokine receptor type 4 (CXCR4) expressed as one of the signature genes on bone-seeking breast cancer cells and its ligand stromal-derived-factor-1 expressed by cells within the bone environment including osteoblasts [2, 3]. The adaption of bone metastatic niche involves exchange of factors between breast cancer cells and cells naturally residing in the bone niche. Previous studies have shown that the osteolytic bone lesion, which is induced by metastatic breast cancer cells through secretion of bone catabolic factors including parathyroid hormone-related protein (PTHrP), interleukin (IL)-11, IL-8, and IL-6, in turn exacerbates this tropism by promoting cancer cell growth in the bone via growth factors released from the degraded bone matrix [4, 5].

In addition to cytokines, extracellular vesicles (EVs) that contain biomaterials such as microRNA (miRNA), mRNA, and proteins, including exosomes, are important vehicles for intercellular communication in short or long range [6]. Our lab has previously shown that breast cancer-secreted EVs target endothelial cells and fibroblasts to induce vascular permeability and metabolic reprogramming, respectively [7–9]. Stromal cells in the metastatic niche can also secrete EVs to regulate cancer cells [10]. Bone marrow mesenchymal stem cells secrete miR-23b-containing exosomes, which induce breast cancer dormancy by targeting a cell cycle regulator myristoylated alanine rich protein kinase C substrate (MARCKS) [11]. Interestingly, a recent study found that exosomes also exhibited organotropism, which was regulated by the integrin profile of these exosomes [12].

Human adult bones constantly undergo turnover and remodeling to replace old bones and to repair damaged ones [13]. Osteoclast-mediated bone resorption can be regulated by osteoblasts, which secrete osteoclast differentiation-inducing factor receptor activator of nuclear factor kappa-Β ligand (RANKL) and its decoy receptor osteoprotegerin (OPG) [14]. Conversely, recent studies revealed that osteoclasts could also secrete coupling factors to regulate proliferation, migration and differentiation of osteoblasts during new bone formation [15]. Osteoblasts secrete type I procollagen into the matrix during differentiation and bone formation [16]. A disintegrin-like and metalloprotease with thrombospondin type 1 motif, 2 (ADAMTS2) procollagenase then cleaves and releases the N′-terminus of type I procollagen [17], which is often referred to as P1NP and widely used as a marker for bone formation [18]. Crosslinked type I collagens then constitute 90% of the organic matrix of the bone [16].

The transforming growth factor beta (TGF-β) superfamily has been shown to regulate bone physiology [19]. TGF-β can either enhance or repress osteoclast differentiation dependent on dose and the presence of other cell types [20, 21]. TGF-β1 has also been reported to induce bone formation through regulating bone mesenchymal stem cell migration [22]. Different bone morphogenetic proteins (BMPs) are implicated in the positive or negative regulation of bone formation [23]. Activin A and activin B, which also belong to the TGF-β superfamily, are homodimers of inhibin βA and inhibin βB, respectively [24]. They bind to type II receptors on plasma membrane and subsequently activate type I receptors. Mothers against DPP homolog (SMAD)2 and SMAD3 are then phosphorylated and bind with SMAD4, which translocates to the nucleus and regulates target gene transcription. Inhibins, which are heterodimers of inhibin α and β subunits, usually antagonize the effect of activins. Blockade of activin signaling by a soluble activin receptor type IIA fusion protein can promote osteoblast-mediated bone formation and inhibit breast cancer bone metastasis in a murine model [25], indicating an important role of activins in bone homeostasis and bone metastasis.

Here we set out to understand the function of miR-218, which is detected at a higher level in the sera from stage IV breast cancer patients with bone metastasis and in the EVs of bone-tropic breast cancer cells, in mediating the profound interplay between breast cancer and bone cells. Our data suggest a model through which cancer-derived miR-218 inhibits osteoblast function of collagen deposition though direct targeting of collagen type I alpha 1 chain (COL1A1) and regulation of inhibin βA expression.

## Methods

### Clinical specimens

Human serum specimens were obtained from voluntarily consenting breast cancer patients between February 2006 and December 2011 at the City of Hope National Medical Center (Duarte, CA, USA) under institutional review board-approved protocols. All 47 patients involved in this study had stage IV disease with or without bone metastases at the time metastatic disease was diagnosed. Among them, 33 patients had bone metastases, in most cases with concurrent metastases to other organs, whereas the other 14 patients had distant metastases to other organs without the involvement of bone. The two groups exhibited balanced age, tumor subtype, and sample collection time. Serum specimens examined in this study were collected at the time metastasis was initially diagnosed or the earliest draw available. Clinical characteristics are summarized in Additional file 1: Table S1. Trizol LS reagent (Thermo Fisher Scientific; Waltham, MA, USA) was used to extract total RNA from ~ 0.5 ml of serum; RNA pellet was dissolved in 10 μl of RNase-free water and subjected to Solexa sequencing and RT-qPCR as previously described [26].

### Cells

Human breast cancer cell line MDA-MB-231, MCF-7, human non-cancerous mammary epithelial cell line MCF10A, and mouse preosteoblast cell line MC3T3-E1 were obtained from American Type Culture Collection (Manassas, VA, USA) and cultured in the recommended media. The bone-tropic subline of MDA-MB-231 was a kind gift from Dr. T. Yoneda. All cells used herein were tested to be free of mycoplasma contamination and authenticated by using the short tandem repeat profiling method. MDA-MB-231 cells were stably transduced with control and miR-218 lentiviral constructs purchased from GeneCopoeia (Rockville, MD, USA). miRIDIAN miR-218 mimic and the corresponding negative control were purchased from GE Dharmacon (Lafayette, CO, USA). LNA oligonucleotides against miR-218 and control were purchased from Exiqon (Woburn, MA, USA). Cell transfection, reporter assays, production of viruses, infection and selection of transduced cells, as well as flow cytometry for cell characterization, were carried out as previously described [7–9, 27]. Osteoblast differentiation was induced by 50 μg/ml L-ascorbic acid, 10 mM β-glycerolphosphate, and 0.1 μM dexamethasone for 16–21 days with medium change every 3–4 days. Conditioned medium was collected by passing through 0.45 μm filters. Conditioned medium concentration was performed using Vivaspin 20 columns from Sartorius (Bohemia, NY, USA).

### Primary bone marrow cell isolation and induction of osteoclast differentiation

One million bone marrow cells isolated from C57BL/6 mice were bulk cultured in a six-well plate and 40 ng/ml M-CSF was added to induce adherence for 3 days. Osteoclast differentiation was induced by adding 40 ng/ml M-CSF and 100 ng/ml RANKL (R&D Systems; Minneapolis, MN, USA) and culturing the cells for 3–7 days. Tartrate-resistant acid phosphatase (TRAP) staining was performed using an Acid Phosphatase, Leukocyte (TRAP) Kit (Sigma-Aldrich; St. Louis, MO, USA).

### Constructs

PCR primers 5′- GATCAACTCGAGGTACACGGTGGGCTGAGTA and 5′- GATCAAGCGGCCGCCCGTGGCACTCAATCTTTTA were used to clone the wild-type 3’ untranslated region (UTR) of human INHBB. The PCR-amplified fragments were digested with XhoI and NotI and then inserted into the same sites of psiCHECK-2 reporter vector (Promega; Madison, WI, USA) downstream of the Renilla luciferase gene. PCR primers 5′- AATTGCGCCTTCCGAGCACACATAA**CTCAGAT**AAGACAGAGACGCAGAGA and 5′- CTCTCTCTCTCTGCGTCTCTGTCTT**ATCTGAG**TTATGTGTGCTCGGAAGG (mutated nucleotides underlined) were used to clone the miR-218-site-mutant of INHBB 3’UTR. Similarly, human Yin and Yang 1 (YY1) 3’UTR was cloned into psiCheck-2 vector using primers 5’-GATCAACTCGAGTTCTCGACCACGGGAAGCA, 5’-GATCAAGCGGCCGCTGAAATTAAGCTACTGGCACTCAA, and mutagenesis primers 5’-AAGAATATGGCAGAACAAGATCTGT**CTCAGAT**GTCTTATTTTCTTTTGTT and 5’-TCTGGACAACAAAAGAAAATAAGAC**ATCTGAG**ACAGATCTTGTTCTGCCA. The YY1 overexpression plasmid was constructed by cloning the full-length YY1 cDNA amplified by primers 5’-GATCAGAATTCATGGCCTCGGGCGACA and 5′- AATAGGATCCTCACTGGTTGTTTTTGGCCT from MDA-231 cells, and inserting the cDNA into the EcoRI/BamHI sites of pSG5 vector. All constructs were verified by sequencing.

### EV purification and characterization

EVs secreted by MDA-MB-231 and derived cell lines were prepared as previously reported [7–9]. Conditioned medium was first collected after incubating cells in growth medium containing 10% EV-depleted FBS (prepared by overnight ultracentrifugation of medium-diluted FBS at 100,000 × g at 4 °C) for 48 h, and pre-cleared by centrifugation at 500 × g for 15 min and then at 10,000 × g for 20 min. EVs were isolated by ultracentrifugation at 110,000 × g for 70 min, and washed in PBS using the same ultracentrifugation conditions. When indicated, DiI (1,1′-Dioctadecyl-3,3,3′,3′-tetramethylindocarbocyanine perchlorate; Sigma-Aldrich) was added into the PBS at 1 μM and incubated for 20 min before the washing spin, followed by an additional wash to remove the excess dye. The pelleted EVs were resuspended in ~ 100 μl of PBS for cell treatment. For cell treatment, 2 μg of EVs (equivalent to those collected from ~ 5 × 10^6^ producer cells) based on protein measurement using Pierce™ BCA protein assay kit (Thermo Fisher Scientific) were added to 2 × 10^5^ recipient cells. For EV characterization, EVs were subjected to nanoparticle tracking analysis using a NanoSight NS300 (Malvern; Westborough, MA,USA), or further fractionated by gradient separation following a modified protocol [28]. EVs isolated by ultracentrifugation were loaded onto a 5-step OptiPrep (Sigma-Aldrich) gradient consisted of 40, 30, 20, 10, and 5% iodixanol in 20 mM Hepes (pH 7.2), 150 mM NaCl, 1 mM Na_3_VO_4_, and 50 mM NaF. After centrifugation in a SW 40 Ti rotor (Beckman Coulter; Indianapolis, IN, USA) at 110,000 × g at 4 °C for 16 h, 12 1-mL fractions were collected and washed in PBS by another spin at 110,000 × g for 70 min before Western analysis and RNA extraction for RT-qPCR.

### RNA extraction and quantitative reverse transcription PCR

These procedures were performed as described previously [7–9]. Primers used in RT-qPCR are indicated in Additional file 2: Table S2. The miR-218, miR-140-3p (as internal control for miR-218 in EVs and sera), and U6 primers (as internal control for intracellular miR-218) were purchased from Qiagen (Valencia, CA, USA). An annealing temperature of 57.5 °C was used for all primers.

### Western blot analysis

Protein extracts were separated by SDS-PAGE. Protein detection was performed using the following antibodies: Collagen alpha-1(I) chain carboxy-telopeptide (LF68) (Kerafast; Boston, MA, USA, ENH018), Inhibin βA (Novus, NBP1–30928), Inhibin βB (Thermo Fisher Scientific, PA5–28814), Inhibin α (Santa Cruz Biotechnology; Dallas, TX, USA, SC-22048), YY1 (Abcam; Cambridge, MA, USA, ab109228), Phospho-Smad2 (Ser465/467) (Cell Signaling Technology; Danvers, MA, USA, 3108S), SMAD2 (Cell Signaling Technology, 3122S), Phospho-Smad3 (Ser423/425) (Cell Signaling Technology, 9520S), SMAD3 (Cell Signaling Technology, 9523S), tissue inhibitor of metalloproteinases 3 (TIMP3) (Abcam, ab155749), β-actin (Sigma-Aldrich, A1978), as well as anti-rabbit, anti-mouse, and anti-goat HRP-conjugated secondary antibodies (Santa Cruz Biotechnology).

### ELISA

Bone formation marker P1NP was detected by a rat/mouse P1NP enzyme immunoassay kit (Immunodiagnostic Systems; Boldons, UK). Bone resorption marker C-terminal telopeptide (CTX-1) was detected by a RatLaps EIA kit (Immunodiagnostic Systems).

### Animals

All animal experiments were approved by the institutional animal care and use committee at the Beckman Research Institute of the City of Hope. Female NOD/SCID/IL2Rγ-null (NSG) mice of 6-month-old were used in this study. EVs from ~ 10^7^ cancer cells were used to treat mice through tail vein injection with 27G needles twice a week for 4 weeks. Serum was collected 1 week after the last EV injection via retro-orbital bleeding.

### smRNA-seq and RNA-seq

Illumina sequencing was performed by the City of Hope Integrative Genomics Core using RNA samples from patient sera, EVs from parental and bone-tropic MDA-231, and MDA-231 transduced with miR-218-overexpressing or control vector. For smRNA-seq, each serum sample was independently subjected to library preparation and deep sequencing. All small RNAs of 15–52 nts were selected and sequenced using the Hiseq 2500 system, following the manufacturer’s protocol (Illumina; San Diego, CA, USA). Raw counts were normalized by trimmed mean of M value (TMM) method and differentially expressed miRNAs between patients with and without bone metastasis or between different cell lines were identified using Bioconductor package “edgeR”. The miRNAs will be regarded as differentially expressed when their *P* values were less than 0.05, minimum expression value more than 50 and log2 fold change more than 1. For RNA-seq, poly(A) RNA was enriched and reverse-transcribed into cDNA, followed by end repair, A-tailing, and linker ligation. The ligated material was amplified by PCR and then analyzed on a HiSeq2500 (Illumina) for parallel sequencing. Sequences were aligned to human genome assembly hg19. Quantification of RefSeq mRNAs was performed using customized R scripts. Counts were normalized by TMM method and differential expression analysis was performed using Bioconductor package “edgeR”.

### Statistics

All quantitative data are presented as mean ± standard deviation (s.d.) unless stated otherwise. Two-sample two-tailed Student *t* tests were used for comparison of means of quantitative data between two groups. For multiple independent groups, one-way ANOVA with post hoc Tukey tests were used. Values of *P* < 0.05 were considered significant. Sample size was generally chosen based on preliminary data indicating the variance within each group and the differences between groups. All samples/animals that have received the proper procedures with confidence were included for the analyses. For experiments in which no quantification is shown, images representative of at least three independent experiments are shown.

## Results

### miR-218 is associated with breast cancer bone metastasis

**Fig. 1:**
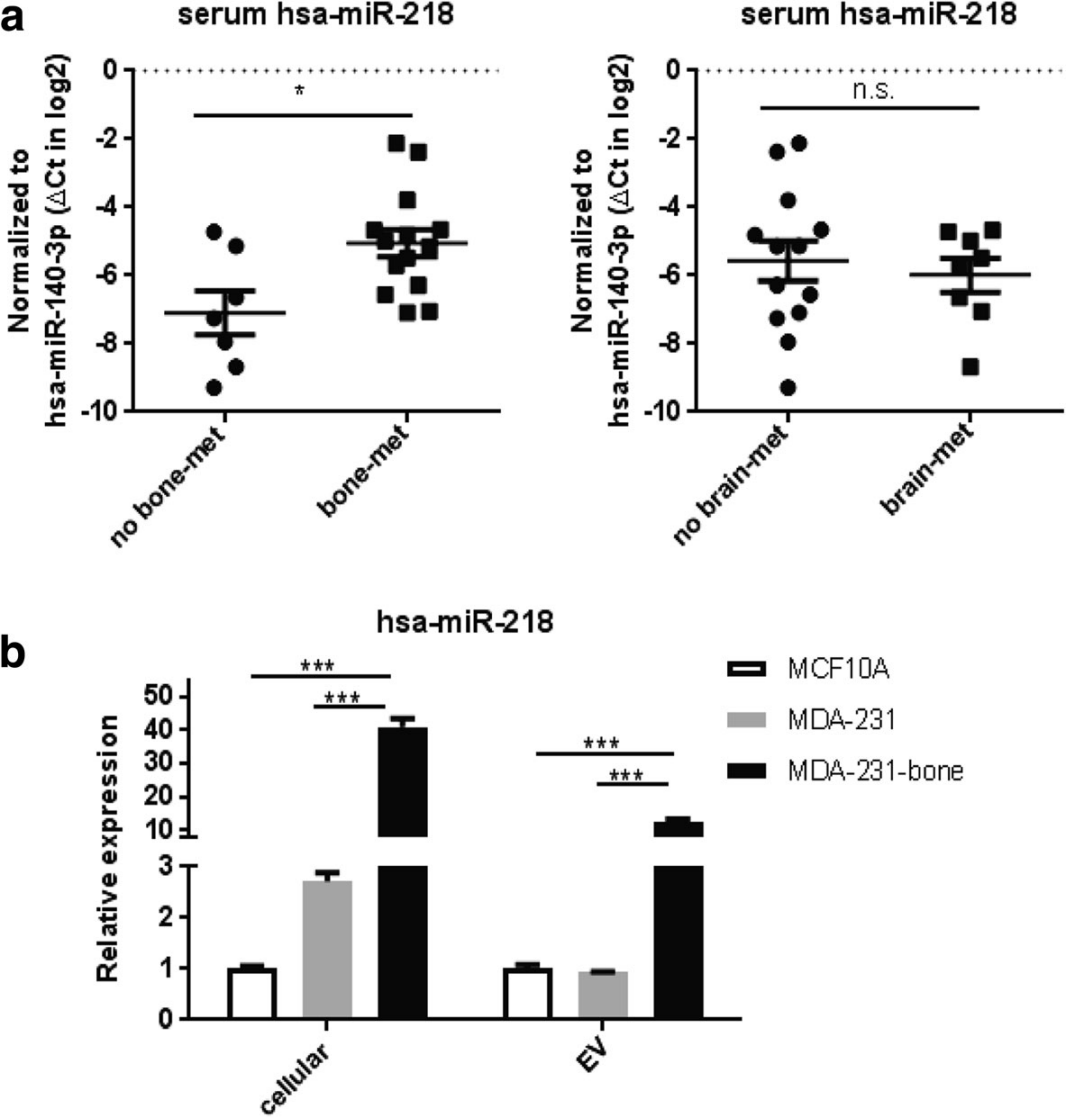
miR-218 is associated with breast cancer bone metastasis. **a** Relative RNA level of miR-218 normalized to miR-140-3p in sera from stage IV breast cancer patients with or without bone metastases (bone-met) (*left panel*), or patients with or without brain metastases (brain-met) (*right panel*). *P < 0.05. n.s. not significant. **b** Relative RNA level of intracellular and EV miR-218 normalized to U6 and miR-140-3p, respectively, in indicated cell lines. ***P < 0.001

To search for miRNAs that might be functionally relevant to breast cancer bone metastasis, we obtained sera from 47 stage IV breast cancer patients with (*n* = 33) or without (*n* = 14) bone metastases and performed small RNA sequencing. In addition, to identify miRNAs characteristically secreted by bone-metastasizing breast cancer cells, we profiled the miRNAs in the EVs secreted by the metastatic breast cancer cell line MDA-MB-231 (MDA-231) as well as its bone-seeking variant designated as MDA-231-bone [29]. hsa-miR-218-5p (miR-218) was significantly higher in the sera from patients with bone metastases compared to those without (Additional file 3: Table S3), and was > 5-fold higher in the EVs from MDA-231-bone cells compared to those from parental MDA-231 (Additional file 4: Table S4). These results were confirmed by qRT-PCR using a selected internal reference miR-140-3p, which was consistent among all serum samples tested and between EVs from the two cell lines based on the smRNA-seq data (Fig. 1a and b). In contrast, levels of circulating miR-218 were not significantly different when the same cohort of patients was stratified by the presence or absence of brain metastases (Fig. 1a, right panel). In addition, miR-218 was also expressed at a higher intracellular level in the bone-tropic MDA-231 cells compared to parental MDA-231 and the non-cancerous mammary epithelial cells MCF10A (Fig. 1b). Thus, we focused on miR-218 in the subsequent studies for its potential role in breast cancer bone metastasis.

### Cancer-secreted miR-218 directly targets *Col1a1* in preosteoblasts and decreases type I collagen secretion by differentiated osteoblasts

**Fig. 2:**
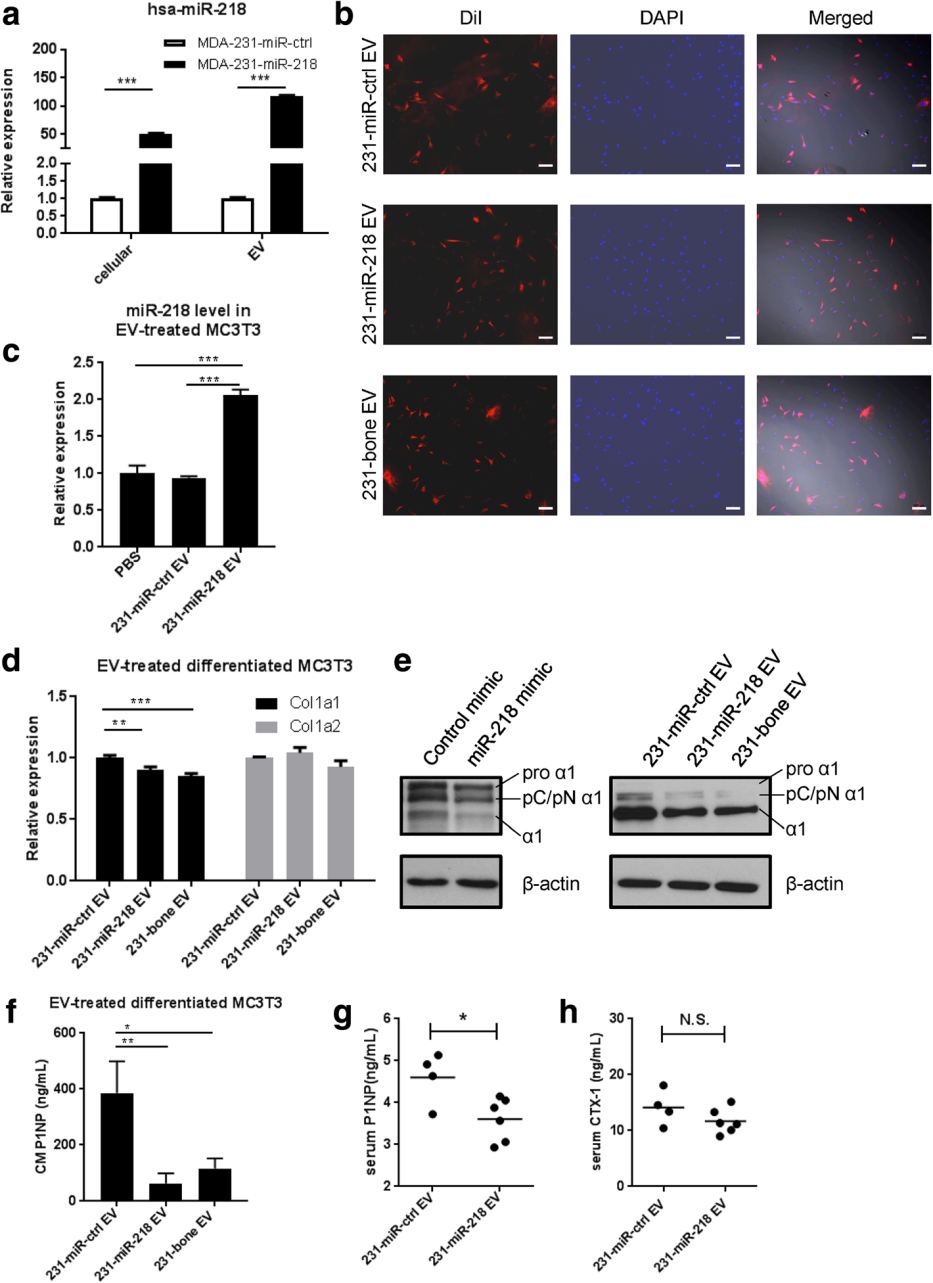
EV miR-218 directly targets Col1a1 and downregulates type I collagen in differentiated MC3T3. **a** Relative RNA level of intracellular and EV miR-218 normalized to U6 and miR-140-3p, respectively, in indicated cell lines. ***P < 0.001. **b** DiI-labeled EV was used to treat MC3T3 cells seeded in a chamber slide for 48 h. Cells were fixed and imaged with fluorescent microscope Zeiss Observer II. Scale bar indicates 100 μm. **c** Relative RNA level of miR-218 normalized to U6 in MC3T3 cells treated with indicated EV or PBS for 24 h in the presence of 10 μM DRB (5,6-dichloro-1-β -D-ribofuranosylbenzimidazole). **d** Relative RNA level of Col1a1, Col1a2 normalized to Actb in differentiated MC3T3 cells treated with indicated EVs. Osteoblast differentiation medium with EV was replenished every 3 to 4 days for 16 days. ***P < 0.001. **e** Western blot analyses of type I collagen in the CM from EV-treated differentiated MC3T3 at day 18. CM of miR-218 mimic-transfected differentiated MC3T3 was also examined. The top band (pro α1), second band (pC/pN α1) and third band (α1) indicates type I procollagen, type I procollagen with either N′ terminus or C′ terminus cleaved, and mature type I collagen with both N′ and C′ termini cleaved, respectively. Cellular β-actin was used as control. **f** P1NP in CM collected from EV-treated differentiated MC3T3 at day 3 was measured by ELISA. *P < 0.05. **g** and **h** Bone formation marker P1NP (**g**) and bone resorption marker CTX-1 (**h**) in the sera from EV-treated mice were measured by ELISA. *P < 0.05; n.s. not significant

To determine the specific effects of miR-218 we established a stable cell line of MDA-231 that overexpresses miR-218 (MDA-231-miR-218) or control vector (MDA-231-miR-ctrl) (Fig. 2a). Compared to the control cells, the miR-218-overexpressing cells also secreted a higher level of miR-218 into the EVs. Upon density gradient fractionation of the EVs, miR-218 was found to be enriched in the fractions containing exosomes and with a density between ~ 1.10 and ~ 1.145 g/mL (Additional file 5: Figure S1). When fluorescently labeled EVs from MDA-231-miR-ctrl, MDA-231-miR-218, and MDA-231-bone were added to preosteoblast cells MC3T3, a high EV uptake efficiency was observed with all types of EVs (Fig. 2b). We also detected transfer of miR-218 into recipient MC3T3 cells upon EV treatment in the presence of an RNA polymerase II inhibitor DRB to block endogenous miR-218 transcription (Fig. 2c). *COL1A1* has been reported as a direct target of miR-218 in gastric cancer cells and human stellate cells [30, 31]. We found that *Col1a1*, but not collagen type I alpha 2 chain (*Col1a2*), was significantly downregulated by EVs from the two high-miR-218-secreting cell lines at the mRNA level (Fig. 2d), and that this effect on type I collagen expression was more dramatic at the protein level (Fig. 2e). Consistent with the lower levels of all forms of type I collagen, the bone formation marker P1NP was also decreased in the conditioned medium (CM) from differentiated MC3T3 treated with high-miR-218 EVs (Fig. 2f). To test the effect of secreted miR-218 in vivo, we injected EVs from MDA-231-miR-ctrl or MDA-231-miR-218 into NSG mice through the tail vein twice a week for 4 weeks. The bone formation marker P1NP was significantly decreased in the serum from mice treated with MDA-231-miR-218 EVs, whereas the bone resorption marker CTX-1 was not affected (Fig. 2g and h). Taken together, cancer-secreted miR-218 encapsulated in the EVs downregulated type I collagen expression and deposition by osteoblasts. In comparison, breast cancer-secreted EVs were found incapable of regulating osteoclast differentiation (Additional file 6: Figure S2).

### miR-218 regulates the expression of inhibin β subunits in breast cancer cells

**Fig. 3:**
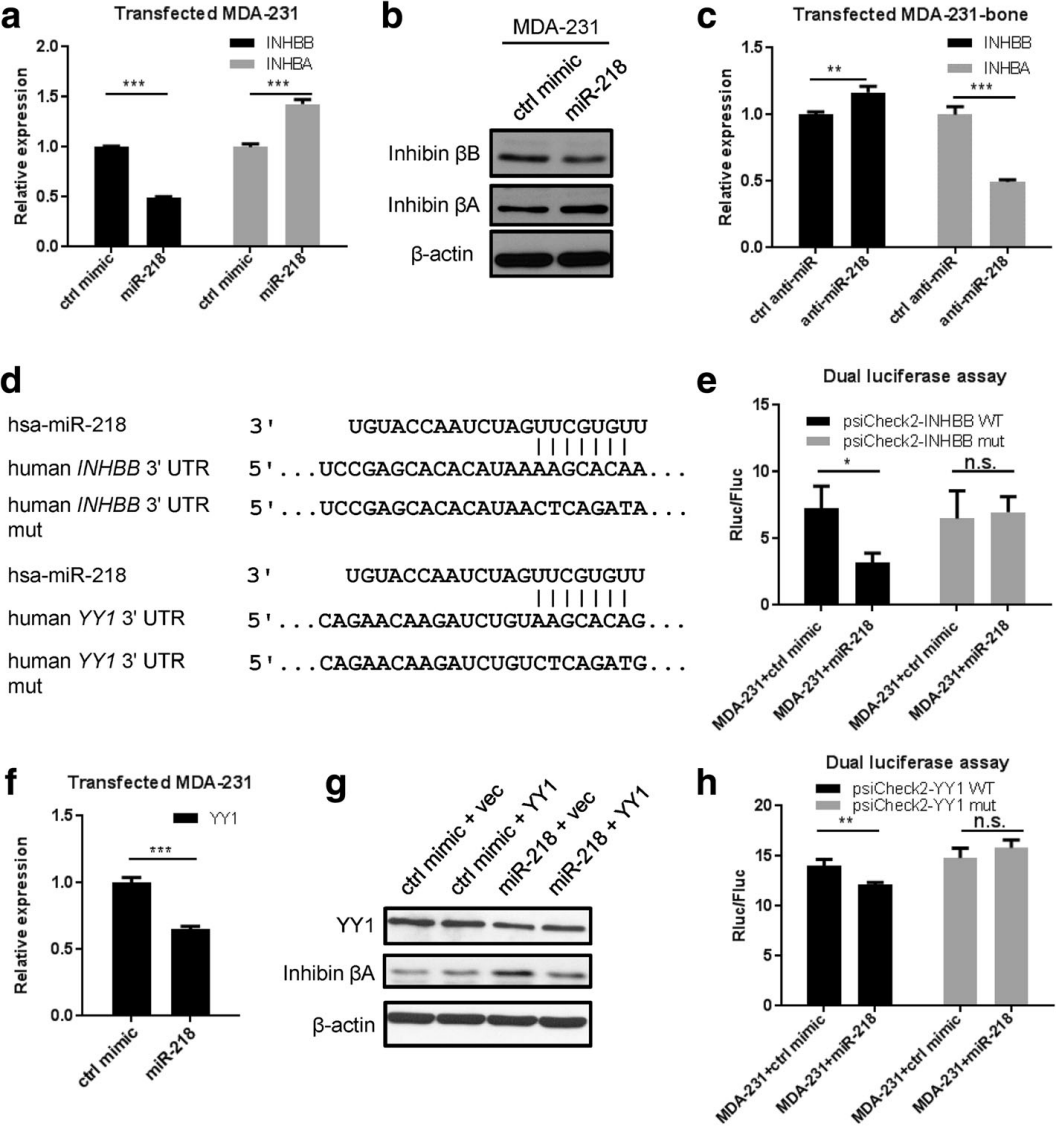
miR-218 directly targets INHBB and YY1, and increases INHBA expression. **a** Relative RNA level of INHBB and INHBA normalized to 18S in MDA-231 transfected with miR-218 or control miRNA mimic at 24 h and 72 h, respectively. ***P < 0.001. **b** Western blot analyses of inhibin βB and inhibin βA in mimic-transfected MDA-231 cells at 48 h. **c** Relative RNA level of INHBB and INHBA normalized to 18S in MDA-231-bone transfected with anti-miR-218 or control antagomiR at 24 h and 48 h, respectively.. **P < 0.01; ***P < 0.001. **d** Sequence alignment of miR-218 and its predicted targets INHBB and YY1. **e** psiCheck2 reporter plasmids and miR-218 mimic or its corresponding control were transfected into indicated cell lines. Renilla and firefly luciferase activity was measured at 48 h. *P < 0.05; n.s. not significant. **f** Relative RNA level of YY1 normalized to 18S in mimic-transfected MDA-231 cells at 24 h. ***P < 0.001. **g** Western blot analyses of YY1 and inhibin βA in MDA-231 cells co-transfected with miR-218 mimic and YY1-overexpressing plasmid, or the corresponding controls at 72 h. **h** psiCheck2 reporter plasmids and miR-218 mimic or its corresponding control vector were transfected into MDA-231 cells. Renilla and firefly luciferase activity was measured at 48 h. **P < 0.01; n.s. not significant

To identify other genes regulated by miR-218, we performed RNA-seq to compare the gene expression profile in MDA-231-miR-ctrl and MDA-231-miR-218 cells. Among the genes showing significantly different expression between the two cell lines, we found that inhibin beta A subunit (INHBA) was upregulated and inhibin beta B subunit (INHBB) downregulated by miR-218 overexpression (Additional file 7: Table S5). This was also observed at both mRNA and protein levels in parental MDA-231 cells upon transient transfection of a miR-218 mimic (Fig. 3a and b). Similar results were observed in another breast cancer cell line MCF-7, where miR-218 also upregulated INHBA but downregulated INHBB expression (Additional file 8: Figure S3a). In contrast, transfection of an antagomiR against miR-218 into MDA-231 bone cells led to a lower mRNA level of INHBA and a higher level of INHBB (Fig. 3c). Since INHBB is predicted to be a direct target of miR-218 by TargetScan (Fig. 3d), we performed dual luciferase reporter assay using psiCheck2 vector to confirm that the 3’UTR of INHBB containing the wild-type, but not mutated, miR-218 binding site responded to miR-218 (Fig. 3e). In search of the mechanism through which INHBA was upregulated by miR-218, we focused on *YY1*, a transcriptional repressor that is also a putative miR-218 target (Fig. 3d) and has been associated with Inhba expression in ovaries [32]. *YY1* mRNA was downregulated by miR-218 in MDA-231 cells (Fig. 3f). Ectopic expression of YY1 abolished the effect of miR-218 on inducing inhibin βA (Fig. 3g). Furthermore, dual luciferase assay revealed that *YY1* was directly targeted by miR-218 through the predicted binding site in the 3’UTR (Fig. 3h). Thus, miR-218 regulates the expression of two TGFβ superfamily cytokines in breast cancer cells by directly targeting *YY1* and *INHBB*.

### Cancer-secreted inhibin βA regulates SMAD2/3 signaling differently in cancer and preosteoblast cells

**Fig. 4:**
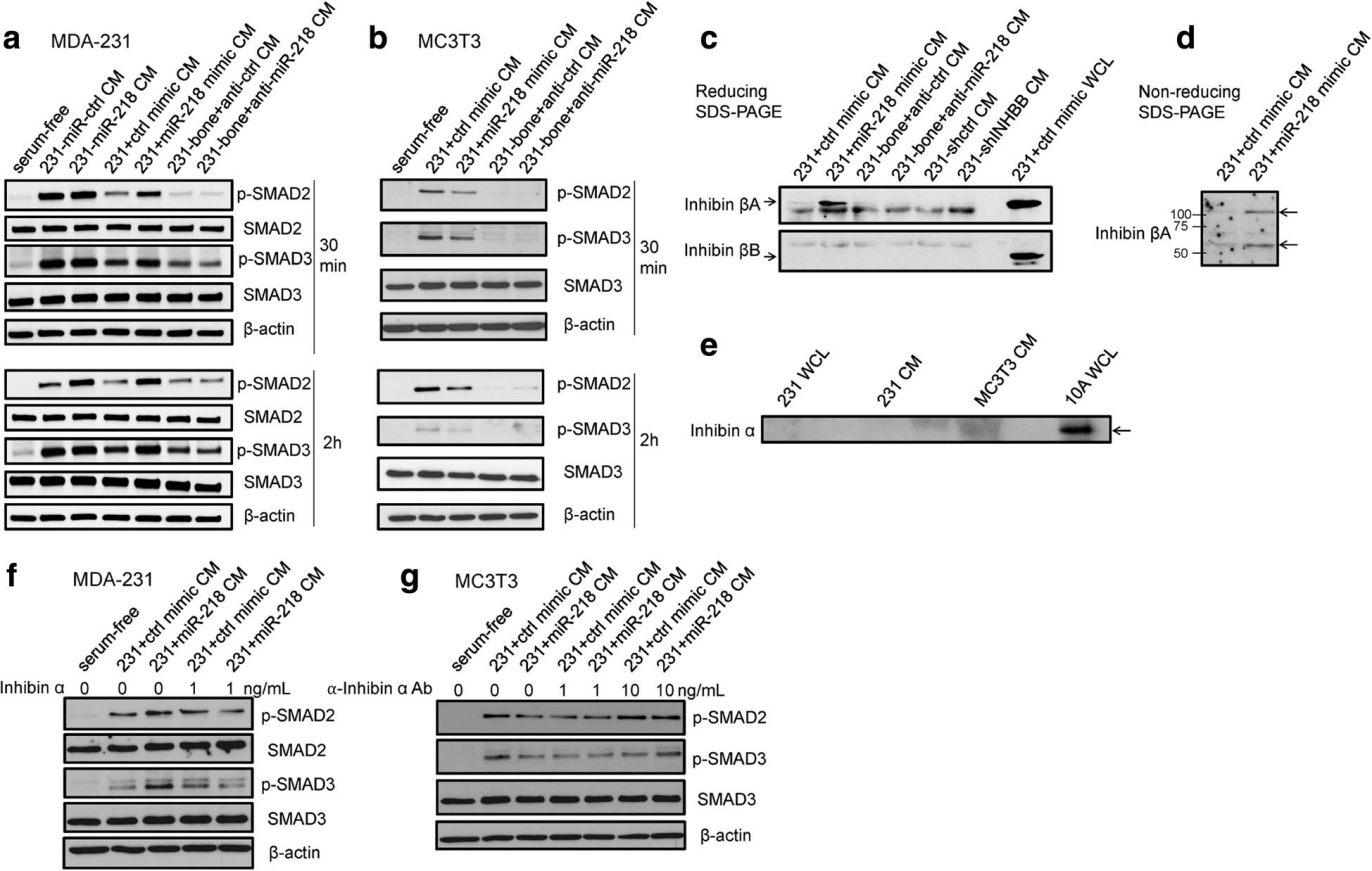
miR-218-regulated inhibin βA affects SMAD2/3 signaling in a cell-dependent manner. **a** and **b** Western blot analyses of phospho-SMAD2/3 in MDA-231 (**a**) or MC3T3 (**b**) that was serum-starved overnight and treated with EV-depleted CM collected from indicated cells for 30 min or 2 h. CM producing cell lines were transfected, PBS washed 48 h after transfection and then incubated with serum-free medium overnight before CM collection and EV depletion by ultracentrifugation. **c** Western blot analyses of inhibin βA, inhibin βB, and inhibin α in CM from indicated cell lines that was concentrated with medium concentrator columns. *Arrows* indicate the position of antigen. Control mimic-transfected MDA-231 whole cell lysate (WCL) was used as positive control for inhibin βA and inhibin βB. **d** Western blot analyses of inhibin βA monomer or dimer (activin A) in CM from indicated cell lines under non-reducing condition and without boiling the samples. *Bottom* and *top arrows* indicate inhibin βA monomer and dimer, respectively. **e** Western blot analyses of inhibin α in MC3T3 CM concentrated from 20 ml CM. WCL of MCF10A was used as a positive control. **f** and **g** Western blot analyses of phospho-SMAD2/3 in MDA-231 (**f**) or MC3T3 (**g**) that was serum-starved overnight and treated with CM collected from indicated cells for 30 min or 2 h. For MDA-231 cells (**f**), recombinant inhibin α protein was added to CM as indicated. For MC3T3 cells (**g**), anti-inhibin α antibody was added to CM as indicated

We continued to test how miR-218-mediated differential expression of inhibin β subunits in breast cancer cells potentially influences the surrounding cancer cells and preosteoblasts to respectively establish autocrine and paracrine signaling. We treated serum-starved MDA-231 or MC3T3 with EV-depleted CM collected from miR-218-transfected MDA-231 cells or anti-miR-218-transfected MDA-231-bone cells. To our surprise, CM from miR-218-overexpressing breast cancer cells induced SMAD2/3 phosphorylation in MDA-231 cells (Fig. 4a), whereas the same CM treatment to MC3T3 cells suppressed the level of phospho-SMAD2/3 (Fig. 4b). Similarly, CM from miR-218-overexpressing MCF-7 cells also induced SMAD2 phosphorylation in untransfected MCF-7 cells (Additional file 8: Figure S3b). Conversely, anti-miR inhibition of miR-218 in CM-producing MDA-231-bone cells led to reduction of SMAD2/3 phosphorylation in cancer cells (Fig. 4a) and de-repression of SMAD2/3 signaling in preosteoblasts (Fig. 4b) at the 2-h time point.

To explain the differential response of SMAD signaling in cancer or bone stromal cells to CM from breast cancer cells with varying levels of miR-218, we concentrated the CM from MDA-231 cells and found that the inhibin βA subunit was secreted at a higher level by miR-218-transfected MDA-231 cells compared to control group while inhibin βB was undetectable in the concentrated CM (Fig. 4c). Moreover, we also detected increased secretion of activin A in the CM from MDA-231 cells transfected with miR-218 (Fig. 4d). Interestingly, concentrated CM from MC3T3 preosteoblast cells contained inhibin α subunits (Fig. 4e), which was not expressed by MDA-231 or MCF-7 breast cancer cells (Additional file 7: Table S5, Fig. 4e, Additional file 8: Figure S3c). We thereby hypothesized that the presence of inhibin α in the environment led to a functional switch of cancer-secreted inhibin βA from activin-mediated SMAD2/3 activation to inhibin-mediated suppression. Indeed, addition of recombinant inhibin α into the CM from miR-218-overexpressing cells reversed the activation of SMAD2/3 phosphorylation in recipient cancer cells (Fig. 4f), whereas adding neutralizing antibody against inhibin α into the CM to treat MC3T3 cells led to de-repression of SMAD2/3 signaling (Fig. 4g). Therefore, secreted inhibin βA, which is upregulated by miR-218 in breast cancer cells, autocrinally activates SMAD signaling in MDA-231 cancer cells but paracrinally represses this pathway in bone stromal cells, due to the different expression status of inhibin α in the environment.

### Paracrinal inhibin βA controls type I collagen processing of differentiating osteoblasts by regulating Timp3 expression

**Fig. 5:**
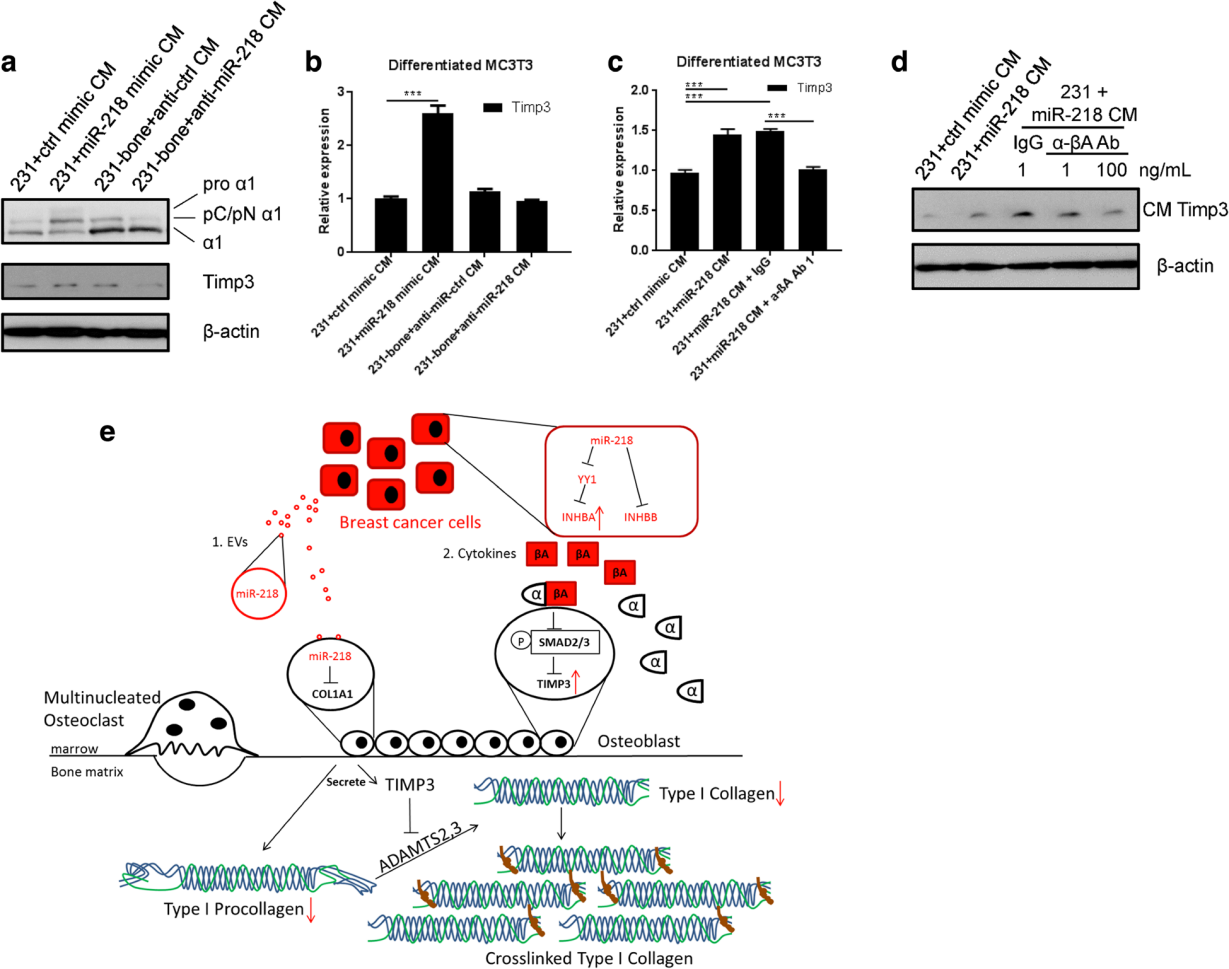
CM from miR-218-overexpressing cells inhibits type I collagen processing in differentiated MC3T3. **a** Western blot analyses of type I collagen and Timp3 in the CM from differentiated MC3T3 treated with indicated EV-depleted CM for 16 days. CM producing cell lines were transfected, PBS washed 48 h after transfection and then incubated with serum-free medium overnight before CM collection and EV depletion by ultracentrifugation. Osteoblast differentiation factors were then added into collected CM, which was used to treated MC3T3 cells and replaced every 3–4 days. Cellular β-actin was used as control. **b** Relative RNA level of Timp3 normalized to 18S in CM-treated differentiated MC3T3 at day 18. ***P < 0.001. **c** Relative RNA level of Timp3 in CM-treated differentiated MC3T3 at day 18. Anti-inhibin βA (1 ng/ml) antibody was added to CM as indicated. ***P < 0.001. **d** Western blot analyses of Timp3 in the CM from differentiated MC3T3 treated with indicated CM with or without anti-inhibin α antibody for 16 days. Cellular β-actin was used as control. **e** Proposed model of bone niche adaptation mediated by miR-218 through direct secretion and targeting of type I procollagen in osteoblasts (1) or regulation of inhibin βA whose secretion in turn blocks procollagen processing by osteoblasts (2)

We next examined the downstream effect of cancer-secreted inhibin βA on preosteoblasts. Tissue inhibitor of metalloproteinases 3 (TIMP3) has been shown to inhibit the activity of ADAMTS2, which is the processing enzyme of type I procollagen during osteoblast differentiation [33]. We found that Timp3 was upregulated in differentiating MC3T3 cells following the treatment with EV-depleted CM from miR-218-overexpressing MDA-231 cells, and was downregulated when miR-218 was inhibited in CM-producing cells (Fig. 5a and b). As a result, the processing of type I procollagen was suppressed during osteoblast differentiation when miR-218 level was high in breast cancer cells, and vice versa (Fig. 5a). To confirm that this effect was caused by higher level of inhibin βA in the CM from miR-218-overexpressing MDA-231 cells, which contributed to the upregulation of Timp3 expression, we added neutralizing antibody against inhibin βA into the CM and detected restoration of Timp3 level in differentiating MC3T3 (Fig. 5c and d). Taken together, we show that paracrinal inhibin βA secretion by breast cancer cells, which is regulated by miR-218, alters the processing of procollagen during osteoblast differentiation through regulating Timp3 expression.

## Discussion

Bone matrix is composed of inorganic and organic materials deposited and mineralized mainly by osteoblasts. Of the organic components of the bone matrix, 90% are type I collagen [16]. During osteoblast differentiation, type I procollagen is expressed and secreted into the bone matrix where ADAMTS2 and BMP1 cleave the N′- and C′-terminus of procollagen, respectively, to generate mature collagens before they are crosslinked and stabilized [17]. Due to the abundance of type I collagen and the importance of its processing in bone formation, P1NP is one of the most commonly used marker for bone formation [18]. Our study identified miR-218 as a miRNA associated with breast cancer bone metastasis, which can inhibit the deposition of type I collagen through two mechanisms (Fig. 5e): (1) miR-218 secretion into the EVs and transfer to osteoblasts during differentiation, where miR-218 directly downregulates COL1A1 expression; (2) direct targeting of YY1 in cancer cells to induce inhibin βA expression and secretion, which then represses procollagen processing by inducing TIMP3, an inhibitor of the N-procollagenase ADAMTS2.

The first mechanism could be initiated at an early tumor stage before bone metastasis occurs, as miR-218-containing EVs secreted by breast cancer cells in situ could travel through the blood stream to the bone environment where miR-218 could exert its effect on osteoblasts after being taken up. The second mechanism would require close proximity of cancer cells and osteoblasts because a previous study has shown that most circulating activins are bound by their endogenous antagonist follistatin [34]. However, several studies found that serum activin A level was elevated in patients with breast cancer bone metastasis [35, 36], indicating a possible long-range effect of activin A on bone cells including osteoblasts. These two mechanisms, acting both distantly and locally, might have synergistic effects on the inhibition of collagen deposition and bone formation, thereby undermining the quality of newly formed bone. Considering the large amount of collagen within the bone environment, the blockade of collagen deposition by miR-218 might require time to take effect on bone formation and breast cancer bone metastasis. Therefore, miR-218 may be insufficient to induce bone metastasis by itself but may facilitate other cancer-autonomous mechanisms to promote breast cancer bone metastasis.

Our study shows that secreted inhibin βA regulates SMAD2/3 signaling in cancer cells and osteoblasts in opposite directions, which is dependent on the absence or presence of inhibin α subunit. We show that inhibin βA, when encountering inhibin α to form inhibin A, inhibits phosphorylation of SMAD2/3 in osteoblasts, and thereby blocks an important function of these cells by inducing the expression and secretion of TIMP3. As for the consequences of SMAD2/3 activation in cancer cells, a study has reported intense immunohistochemical staining of phosphorylated SMAD2 in metastatic breast tumors in the bone and that knockdown of SMAD4, the downstream effector of activated SMAD2/3, was able to decrease bone metastasis of breast cancer in mice [37]. On the other hand, activin A has been shown to inhibit breast cancer proliferation [38]. Therefore, it is possible that the formation of inhibin A in the bone microenvironment, as a result of osteoblast-derived inhibin α, could alleviate or even reverse the growth inhibitory effect of activin A in the outer layer of tumor mass growing in the bone. Together, increased secretion of inhibin βA might promote breast cancer bone metastasis by regulating SMAD signaling differently in cancer and bone stromal cells.

A recent study on miR-218 also implicated this miRNA in breast cancer metastasis [39]. It also found that miR-218 level was higher in breast cancer metastasized to the bone. In consistent with this, we show that miR-218 is not only overexpressed in bone-tropic breast cancer cells, but also more abundant in EVs from these cells as well as in the sera from breast cancer patients with bone metastases. Mechanistically, this previous study found that miR-218 directly targeted Wnt inhibitors to promote PTHrP expression, which in turn stimulated osteoclastogenesis and osteolytic bone lesion. Another study on miR-218 suggested that this miRNA had a role in the induction of osteomimicry phenotype of breast cancer cells [40]. Therefore, it is possible that miR-218 contributes to a pro-metastatic bone environment through regulating the function of both osteoclasts and osteoblasts, thereby tilting the balance towards osteoclast-mediated bone resorption and providing a permissive environment for cancer growth in the bone. Based on the association between miR-218 and breast cancer bone metastasis, it would be of interest to test the possibility of using miR-218 as a diagnostic and/or prognostic marker for the disease.

## Conclusions

In summary, our study showed that miR-218, which exhibited higher levels in the blood of breast cancer patients with bone metastases, and which was overexpressed and highly secreted by bone-tropic MDA-MB-231 breast cancer cells, could contribute to the adaption of bone niche. Cancer cell-secreted miR-218 directly downregulated type I collagen expression by osteoblasts, whereas intracellular miR-218 in breast cancer cells elevated inhibin βA expression, whose secretion in turn inhibited the processing of type I collagen during collagen deposition and osteoblast differentiation. Together, compromised collagen deposition might further enhance the vicious cycle of osteolysis and thereby facilitate other cancer-autonomous mechanisms to promote breast cancer colonization in the bone environment.

## Additional files

Additional file 1: **Table S1.** Clinical characteristics of the patients. (XLSX 23 kb)

Additional file 2: **Table S2.** Sequences of the PCR primers. (XLSX 9 kb)

Additional file 3: **Table S3.** miRNAs detected in patient sera. (XLSX 122 kb)

Additional file 4: **Table S4.** Cellular and EV miRNAs detected in MDA-231-bone and parental MDA-231 cells. (XLSX 128 kb)

Additional file 5: **Figure S1.** EV characterization. **a** EVs pelleted at 110,000 × g were analyzed by nanoparticle tracking analysis. **b** Density measurement and Western blot of EV fractions collected from indicated cell lines to detect EV markers. **c** Density measurement and RT-qPCR of EV fractions collected from indicated cell lines to detect miR-218 levels. (PDF 1060 kb)

Additional file 6: **Figure S2.** Breast cancer-secreted EVs did not regulate osteoclast differentiation. Mouse bone marrow cells were cultured in 40 ng/ml M-CSF for 3 days before EV treatment and further induction of osteoclast differentiation with 40 ng/ml M-CSF and 100 ng/ml RANKL for up to 7 days. **a** Representative TRAP staining images of EV-treated osteoclasts after 7 days of differentiation. **b** Quantitative analysis of TRAP staining in (**a**). Mature osteoclasts were identified as multinucleated TRAP^+^ cells. **c** Relative RNA level of osteoclast differentiation marker genes *Trap* and *Ctsk* normalized to *Rpl19* in primary pre-osteoclast cells treated with indicated EVs and induced for osteoclast differentiation for 5 days. (PDF 318 kb)

Additional file 7: **Table S5.** Gene expression in MDA-231-miR-218 and MDA-231-miR-ctrl cells. (XLSX 1093 kb)

Additional file 8: **Figure S3.** miR-218 regulated inhibin β expression and enhanced SMAD signaling in MCF-7 cells. **a** Western blot analyses of inhibin βB and inhibin βA in miRNA mimic-transfected MCF-7 cells at 48 h after transfection. **b** Western blot analyses of phospho-SMAD2/3 in MCF-7 cells that were serum-starved overnight and then treated with CM collected from indicated cells for 30 min. The CM-producing cells were transfected, PBS washed at 48 h after transfection, and then incubated with serum-free medium overnight before CM collection. **c** Western blot analysis of inhibin α in MCF-7 cells. WCL of MCF10A was used as a positive control. (PDF 145 kb)

## Abbreviations

ADAMTS2: A disintegrin-like and metalloprotease with thrombospondin type 1 motif, 2
BMPs: Bone morphogenetic proteins
CM: Conditioned medium
COL1A1: Collagen type I alpha 1 chain
COL1A2: Collagen type I alpha 2 chain
CTX-1: C-terminal telopeptide
CXCR4: C-X-C chemokine receptor type 4
DiI: 1,1′-Dioctadecyl-3,3,3′,3′-tetramethylindocarbocyanine perchlorate
DRB: 5,6-dichloro-1-β -D-ribofuranosylbenzimidazole
EV: Extracellular vesicle
IL: Interleukin
INHBA: Inhibin beta A subunit
INHBB: Inhibin beta B subunit
MARCKS: Myristoylated alanine rich protein kinase C substrate
M-CSF: Macrophage colony-stimulating factor
MDA-231: MDA-MB-231
miRNA: microRNA
NSG: NOD-scid IL2Rgamma(null)
OPG: Osteoprotegerin
P1NP: Procollagen type 1 amino terminal propeptide
PTHrP: Parathyroid hormone-related protein
RANKL: Receptor activator of nuclear factor kappa-Β ligand
SMAD: Mothers against DPP homolog
TGF-β: Transforming growth factor beta
TIMP3: Tissue inhibitor of metalloproteinases 3
TRAP: Tartrate-resistant acid phosphatase
UTR: Untranslated region
YY1: Yin and Yang 1
