# Supplementary material for: Metastatic breast cancer cells overexpress and secrete miR-218 to regulate type I collagen deposition by osteoblasts
Source: Breast Cancer Res. 2018 Oct 22;20:127. doi: 10.1186/s13058-018-1059-y (PMC6198446; doi:10.1186/s13058-018-1059-y)
Supplement: Supplementary file 5 — Figure S1. EV characterization. a EVs pelleted at 110,000 × g were analyzed by nanoparticle tracking analysis. b Density measurement and Western blot of EV fractions collected from indicated cell lines to detect EV markers. c Density measurement and RT-qPCR of EV fractions collected from indicated cell lines to detect miR-218 levels. (PDF 1060 kb) [file 13058_2018_1059_MOESM5_ESM.pdf]

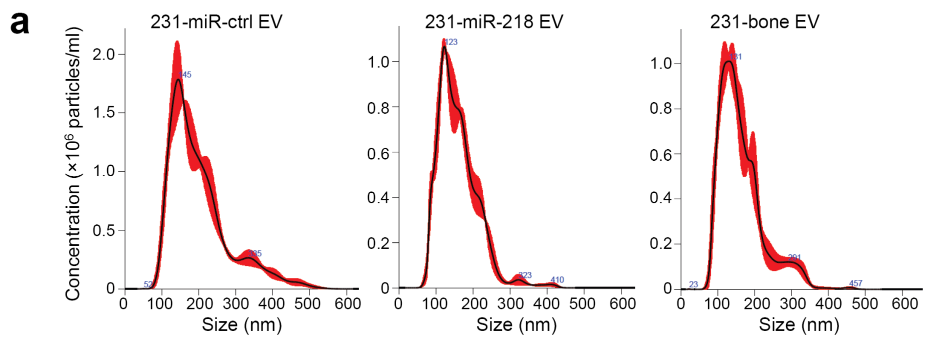

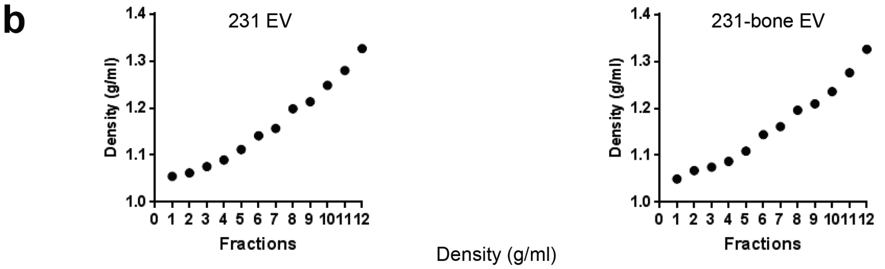

Density (g/ml)

	1	2	3	4	5	6	7	8	9	10	11	12
231 EV	1.0548	1.0622	1.0753	1.0895	1.1117	1.1408	1.1563	1.1983	1.2132	1.2478	1.2795	1.3262
231-bone EV	1.0493	1.0674	1.0748	1.0868	1.1086	1.1440	1.1610	1.1956	1.2095	1.2355	1.2754	1.3252

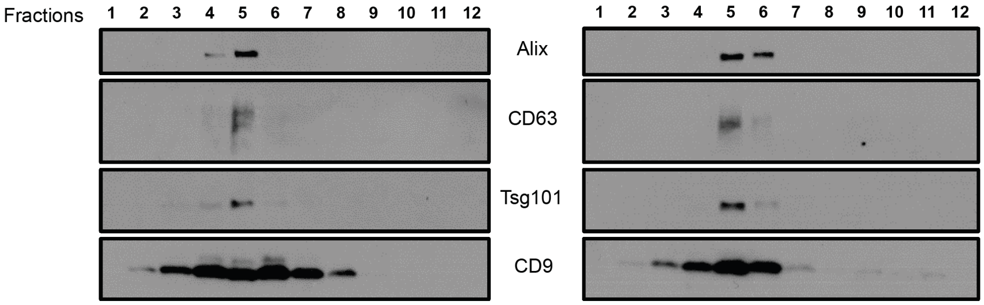

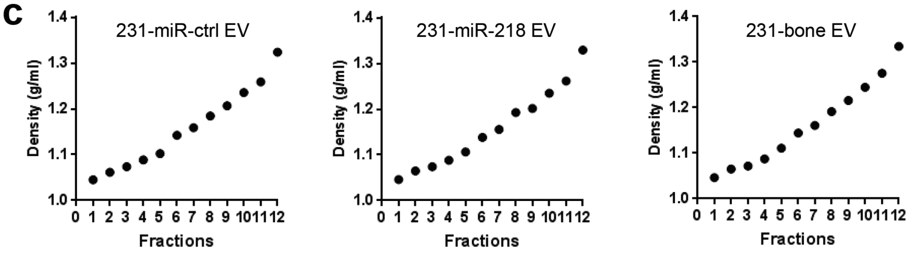

Density (g/ml)

	1	2	3	4	5	6	7	8	9	10	11	12
231-miR-ctrl EV	1.0449	1.0611	1.0735	1.0884	1.1020	1.1419	1.1587	1.1844	1.2064	1.2352	1.2586	1.3233
231-miR-218 EV	1.0457	1.0648	1.0740	1.0881	1.1062	1.1380	1.1553	1.1922	1.2012	1.2347	1.2612	1.3291
231-bone EV	1.0462	1.0651	1.0716	1.0871	1.1110	1.1443	1.1610	1.1912	1.2158	1.2447	1.2754	1.3346

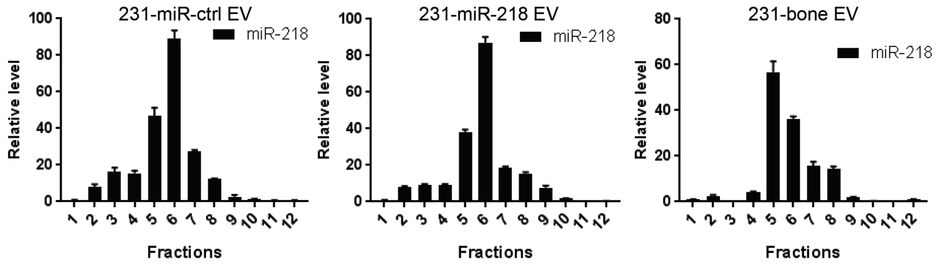

Fig. S1 EV characterization**. a** EVs pelleted at 110,000 ×g were analyzed by nanoparticle tracking analysis. **b** Density measurement and Western blot of EV fractions collected from indicated cell lines to detect EV markers. **c** Density measurement and RT-qPCR of EV fractions collected from indicated cell lines to detect miR-218 levels.
