# Supplementary material for: Metastatic breast cancer cells overexpress and secrete miR-218 to regulate type I collagen deposition by osteoblasts
Source: Breast Cancer Res. 2018 Oct 22;20:127. doi: 10.1186/s13058-018-1059-y (PMC6198446; doi:10.1186/s13058-018-1059-y)
Supplement: Supplementary file 6 — Figure S2. Breast cancer-secreted EVs did not regulate osteoclast differentiation. Mouse bone marrow cells were cultured in 40 ng/ml M-CSF for 3 days before EV treatment and further induction of osteoclast differentiation with 40 ng/ml M-CSF and 100 ng/ml RANKL for up to 7 days. a Representative TRAP staining images of EV-treated osteoclasts after 7 days of differentiation. b Quantitative analysis of TRAP staining in (a). Mature osteoclasts were identified as multinucleated TRAP+ cells. c Relative RNA level of osteoclast differentiation marker genes Trap and Ctsk normalized to Rpl19 in primary pre-osteoclast cells treated with indicated EVs and induced for osteoclast differentiation for 5 days. (PDF 318 kb) [file 13058_2018_1059_MOESM6_ESM.pdf]

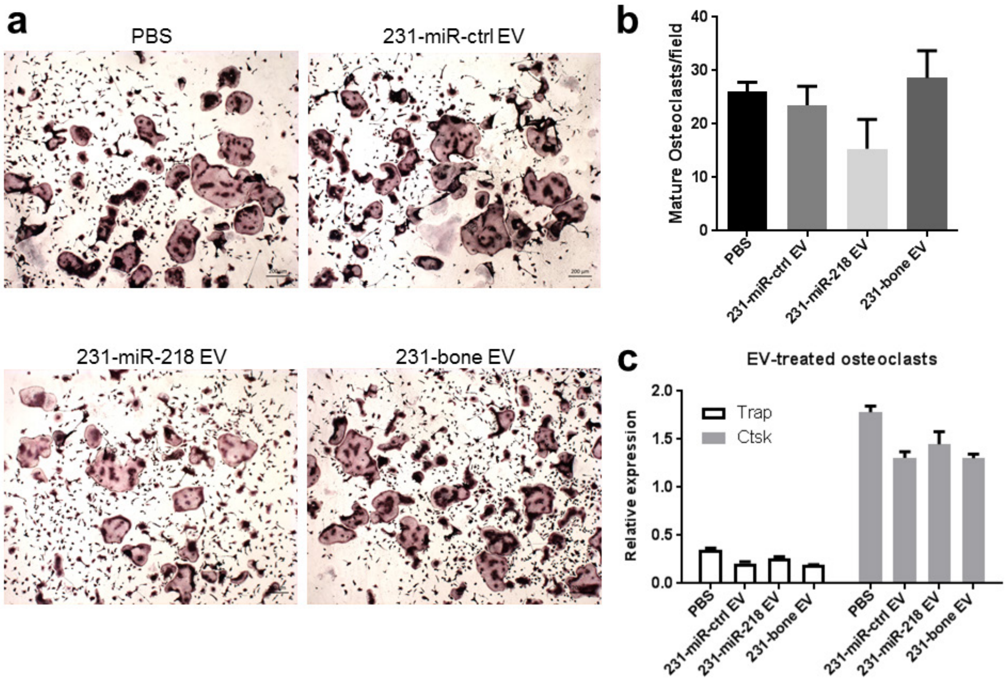

Fig. S2 Breast cancer-secreted EVs did not regulate osteoclast differentiation. Mouse bone marrow cells were cultured in 40 ng/ml M-CSF for 3 days before EV treatment and further induction of osteoclast differentiation with 40 ng/ml M-CSF and 100 ng/ml RANKL for up to 7 days. **a** Representative TRAP staining images of EV-treated osteoclasts after 7 days of differentiation. **b** Quantitative analysis of TRAP staining in (**a**). Mature osteoclasts were identified as multinucleated $TRAP^+$ cells. **c** Relative RNA level of osteoclast differentiation marker genes *Trap* and *Ctsk* normalized to *Rpl19* in primary pre-osteoclast cells treated with indicated EVs and induced for osteoclast differentiation for 5 days.
