# Supplementary material for: Metastatic breast cancer cells overexpress and secrete miR-218 to regulate type I collagen deposition by osteoblasts
Source: Breast Cancer Res. 2018 Oct 22;20:127. doi: 10.1186/s13058-018-1059-y (PMC6198446; doi:10.1186/s13058-018-1059-y)
Supplement: Supplementary file 8 — Figure S3. miR-218 regulated inhibin β expression and enhanced SMAD signaling in MCF-7 cells. a Western blot analyses of inhibin βB and inhibin βA in miRNA mimic-transfected MCF-7 cells at 48 h after transfection. b Western blot analyses of phospho-SMAD2/3 in MCF-7 cells that were serum-starved overnight and then treated with CM collected from indicated cells for 30 min. The CM-producing cells were transfected, PBS washed at 48 h after transfection, and then incubated with serum-free medium overnight before CM collection. c Western blot analysis of inhibin α in MCF-7 cells. WCL of MCF10A was used as a positive control. (PDF 145 kb) [file 13058_2018_1059_MOESM8_ESM.pdf]

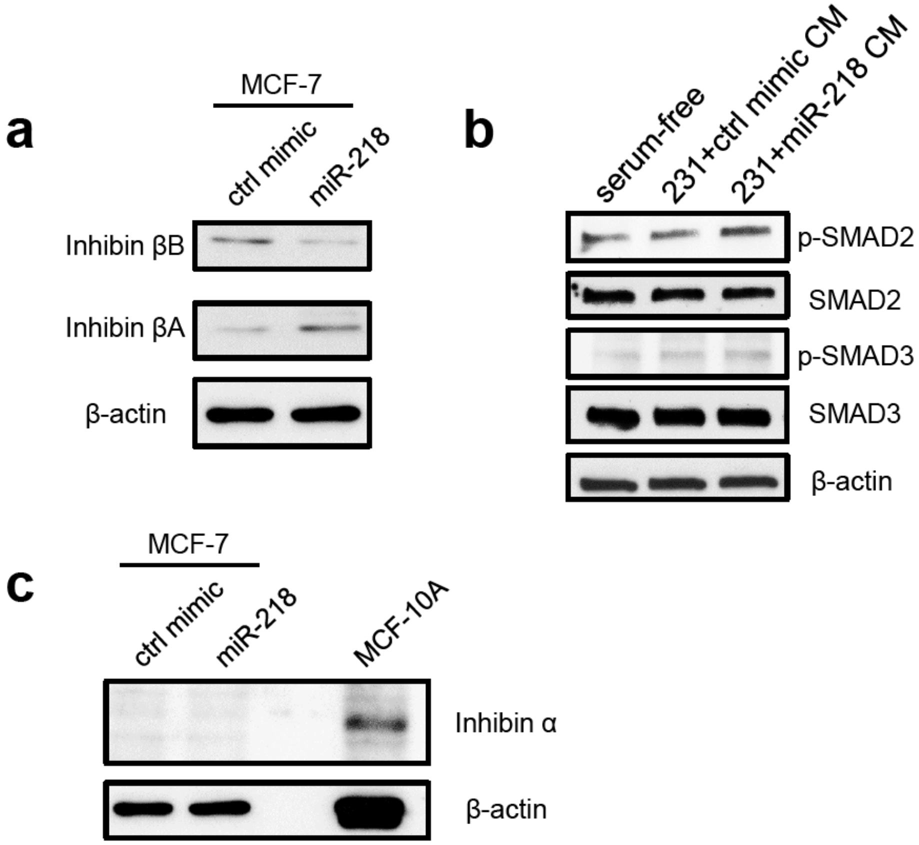

Fig. S3 miR-218 regulated inhibin β expression and enhanced SMAD signaling in MCF-7 cells. **a** Western blot analyses of inhibin βB and inhibin βA in miRNA mimic-transfected MCF-7 cells at 48 h after transfection. **b** Western blot analyses of phospho-SMAD2/3 in MCF-7 cells that were serum-starved overnight and then treated with CM collected from indicated cells for 30 min. The CM producing cells were transfected, PBS washed at 48 h after transfection, and then incubated with serum-free medium overnight before CM collection. **c** Western blot analysis of inhibin α in MCF-7 cells. WCL of MCF10A was used as a positive control.
